# Obstructive sleep apnea and rhonchopathy are associated with downregulation of trefoil factor family peptide 3 (TFF3)—Implications of changes in oral mucus composition

**DOI:** 10.1371/journal.pone.0185200

**Published:** 2017-10-13

**Authors:** Regina Siber-Hoogeboom, Martin Schicht, Sebastian Hoogeboom, Friedrich Paulsen, Maximilian Traxdorf

**Affiliations:** 1 Department of Anatomy II, Friedrich Alexander University Erlangen-Nürnberg (FAU), Erlangen, Germany; 2 Department of Otorhinolaryngology, Head & Neck Surgery, Friedrich Alexander University Erlangen-Nürnberg (FAU), Erlangen, Germany; Technische Universitat Munchen, GERMANY

## Abstract

**Study objectives:**

Trefoil factor family (TFF) peptides belong to the family of mucin-associated peptides and are expressed in most mucosal surfaces. TFF peptides carry out functions such as proliferation and migration enhancement, anti-apoptosis, and wound healing. Moreover, TFFs are associated with mucins and interact with them as “linker peptides”, thereby influencing mucus viscosity.

To test the hypothesis that in rhonchopathy and obstructive sleep apnea (OSA) changes occur in the expression of TFF3 and -2 that could contribute to changes in mucus viscosity, leading to an increase in upper airway resistance during breathing.

**Methods:**

RT-PCR, Western-blot, immunohistochemistry and ELISA were performed to detect and quantify TFF3 and -2 in uvula samples. In addition, 99 saliva samples from patients with mild, moderate or severe OSA, as well as samples from rhonchopathy patients and from healthy volunteers, were analyzed by ELISA.

**Results:**

TFF3 was detected in all uvula samples. Immunohistochemistry revealed a subjectively decreasing antibody reactivity of the uvula epithelia with increasing disease severity. ELISA demonstrated significantly higher TFF3 saliva protein concentrations in the healthy control group compared to cases with rhonchopathy and OSA. Predisposing factors of OSA such as BMI or age showed no correlation with TFF3. No significant changes were observed with regard to TFF2.

**Conclusions:**

The results suggest the involvement of TFF3 in the pathogenesis of rhonchopathy and OSA and lead to the hypothesis that reduction of TFF3 production by the epithelium and subepithelial mucous glands of the uvula contribute to an increase in breathing resistance due to a change in mucus organization.

## Introduction

Snoring (rhonchopathy) is a typical and frequent sound that is generated mainly by soft palate vibrations, besides other anatomic structures, during sleep [1]. In the general population, about 32% of men and 21% of women snore. The snoring induces an impairment of thermal sensitivity, two-point discrimination and histopathological changes in the soft palate [2]. In many cases, snoring is underestimated, leading to the failure to diagnose accompanying pathological conditions such as obstructive sleep apnea (OSA). It is a multifactorial disease in which histological changes, rheological parameters and predisposing factors, such as a high body mass index, age and male sex, play important roles [3–5] that lead to a disturbance of normal sleep and impact the quality of life [6], significantly raising the risk of cardiovascular morbidity [6–8]. The prevention of upper airway collapse is based on non-continuous positive airway pressure (non-CPAP) therapies with oral devices or surgical procedures such as uvulopalatopharyngoplasty (UPPP), accompanied by a reduction of predisposing factors such as adiposity [9–12].

Besides structural changes in the uvula and soft palate [13], the influence of the upper airway lining liquid on surface tension is a factor contributing to upper airway collapsibility [4, 14, 15].

The co-expression of mucins (heavily glycosylated proteins with tandem repeat regions) with trefoil factor family peptides (TFFs), which represent essential elements related to mucus activity, has been conclusively demonstrated [16]. It has been shown that TFFs interact with mucins, thus increasing viscosity. TFF peptides have also been shown to carry out various functions [17] such as proliferation and migration enhancement, anti-apoptosis, and wound healing. However, catabolic functions such as the activation of matrix metalloproteinases and pro-apoptotic effects have also been described beyond the mucosae [18].

As to the interaction of TFFs with mucins, the addition of TFF2 to mucin solutions has been shown to result in significantly increased viscosity and elasticity, whereby the mucin solutions are transformed into a gel-like state [19]. Moreover, the dimeric form of TFF3 has been shown to increase the viscosity of a mucin solution, resulting in a spider's web-like structure, whereas the monomer form of TFF3 has very little effect on viscosity and elasticity [19]. Microarray analysis of TFF3 showed that this peptide improved the mechanical and chemical resistance of mucin [20]. On the other hand, TFF2 showed clearer effects than TFF3, and Thim et al. hypothesized that the different chemical structure might be the cause of the differences in gel-formation [19]. In respiratory mucus, TFF3 is co-localized with the secretory mucins MUC5B, MUC2 and MUC5AC [21]. In human saliva, TFF3 probably co-aggregates with MUC5B and MUC7, the two main mucins in this fluid.

Since OSA is not only associated with epithelial changes [13,14] but also with an increase in airway resistance as well as mouth, throat and nose dryness, and because TFF peptides, in particular TFF3 and -2, have an immense impact on mucus viscosity and rheology (as mentioned above) as well as protective effects such as proliferation and migration enhancement, anti-apoptosis and wound healing, at least in mucosal surfaces, we were keen to learn more about the possible role of TFF3 and -2 in OSA and rhonchopathy. We therefore designed our study to show whether there is a change in TFF3 and TFF2 expression in cases of OSA or rhonchopathy.

## Methods

The investigation of patients and the collection of tissue specimens and saliva samples were performed at the Department of Otorhinolaryngology, Head & Neck Surgery, FAU Erlangen-Nürnberg between April 2012 and February 2013. The study was approved by ethics Committee of the Medical Faculty of the Friedrich Alexander University Erlangen-Nürnberg (FAU). Participant consent was obtained verbal and written. The study did not include minors (age < 18 years).

All 114 participating patients, aged 20 to 83 years, were recruited by the Department of Otorhinolaryngology (ORL), Head and Neck Surgery. 80 patients were men and 34 were women. In addition to a standardized interview, the patients were examined by an otorhinolaryngologist and underwent endoscopy while awake to assess the upper airways. This was followed by cardiorespiratory polysomnography in a sleep laboratory of the ORL Department for the exact classification of sleep-related respiratory disorders. The OSA severity was classified as mild (Apnea Hypopnea Index (AHI) 5-15/h), moderate (AHI 15-30/h) or severe (AHI>30/h) according to the criteria of the American Academy of Sleep Medicine Task Force [13]. The indication for upper airway surgery, including preoperative sleep endoscopy, was established in the context of a primary indication for the treatment of OSA or CPAP non-compliance (secondary or adjuvant indication).

Inclusion criteria for this study were men and women aged 20–83 years with mild, moderate or severe OSA diagnosed by polysomnography. Exclusion criteria were an American Society of Anesthesiologists Classification (ASA) IV/V, central sleep apnea, positive history of sedative, alcohol or addictive drug abuse, propofol allergy or pregnancy.

Cardiorespiratory polysomnography (PSG) was carried out using the diagnostic system SOMNOscreen (SOMNOmedics, Randersacker, Germany). The technical procedure for polysomnographic diagnosis followed the recommendations of the American Academy of Sleep Medicine (AASM) using the standardized technique with an electroencephalogram (EEG; F_4_-M_1_, C_4_-M_1_, O_2_-M_1_), right and left electrooculogram, electromyogram of the mentalis and tibialis muscles, nasal pressure cannula, thoracic and abdominal respiratory effort sensors (inductive plethysmographs), body position sensors, pulse oximetry, snoring microphone, a one-channel ECG and an infrared video recording [22]. The evaluation was performed according to the AASM Criteria (Version 1.0, 2007) and was undertaken by an accredited medical sleep specialist of the German Sleep Society (DGSM) [22].

### Human tissue samples

After polysomnography had confirmed OSA, patients underwent sleep endoscopy to determine the elegibility factor of velar/oropharyngeal obstruction. A standardized propofol-based drug-induced sleep endoscopy (DISE) with TCI and BIS™ (DISE-TCI-BIS) was performed as described previously [23].

Modified UPPP with tonsillectomy was performed based on the procedure described by *Fujita et al*. (1981) [24]. Biopsies were taken of the palatine tonsils, the resected mucosa of the velar webbing (redundant mucosa of the posterior pillar/palatopharyngeal muscle) and the mucosal tip of the resected uvula. Specimens were stored in 4% paraformaldehyde at 5°C.

Human tissue samples were obtained from 15 patients suffering from mild (n = 5), moderate (n = 5) and severe (n = 2) OSA or rhonchopathy (n = 3) and were used for RT-PCR, Western blot analysis and immunohistochemistry.

### Human saliva samples

Saliva samples were obtained by holding Schirmer tear test strips (Optitech eyecare, Allahabad (U.P), INDIA) on the soft palate at the base of the uvula. All strips were immediately stored at -80°C.

Thus a total of 99 saliva samples were collected. In order to extract the saliva, the diagnostic strips were defrosted on ice. Before centrifugation, 100 μl aqua dest. was added to each strip [25], followed by measurement of protein concentration in 2 μl of sample using the Bradford assay. All saliva samples were stored at -20°C.

For TFF3 analysis in saliva, 57 saliva samples were used for ELISA (Table 1, S1 Table). 37 samples from patients with mild (n = 9), moderate (n = 7), or severe (n = 6) OSA were deconstructed as well as 8 samples from rhonchopathy patients (n = 8) and 7 samples from healthy controls (n = 7). The saliva-TFF3 group had a mean age of 47.6 yrs (52.8 yrs mild, 47.4 yrs moderate, 55.7 yrs severe, 35.6 yrs rhonchopathy, 37.5 yrs control) and a mean body mass index of 26.8 kg/m^2^ (26.7 kg/m^2^ mild, 27.5 kg/m^2^ moderate, 32.8 kg/m^2^ severe, 23.3 kg/m^2^ rhonchopathy, 22.9 kg/m^2^ healthy controls) (S2 Table). The total group included 14 smokers and 22 non-smokers (one patient did not answer all questions, so that a total of 36 samples were included).

**Table 1:** Data of AHI, age, BMI, ESS, gender and smoking from healthy controls, rhonchopathy patients, mild, moderate and severe OSA.

	mean AHI	mean age	BMI	ESS	gender		smoking	
					male (%)	female (%)	smoker (%)	non-smoker (%)
**control**								
TFF3	<5	37.5	22.9	4.4	42.9	57.1	0	100
TFF2	<5	43.8	24.8	7.2	40	60	80	20
**rhonchopathy**								
TFF3	<5	35.6	23.3	7.9	50	50	50	50
TFF2	<5	37.8	28.4	12.5	50	50	100	0
biopsy	<5	38.7	29.6	4	100	0	100	0
**mild OSA**								
TFF3	10.2	52.8	26.7	7.8	11.1	88.9	44.4	55.6
TFF2	11.1	55	26.6	5	50	50	50	50
biopsy	11.5	44.2	28.4	12.8	80	20	20	80
**moderate OSA**								
TFF3	22.3	47.4	27.5	7.9	100	0	57.1	42.9
TFF2	20.9	53.1	29.2	8.1	80	20	46.7	53.3
biopsy	22.6	40.6	31	8.6	100	0	40	60
**severe OSA**								
TFF3	59.8	55.7	32.8	12.8	100	0	33.3	66.7
TFF2	55.8	55.7	32.4	9	90	10	90	10
biopsy	33.2	47.5	25.5	8	100	0	100	0

For TFF2 analysis, 42 saliva samples were used for ELISA (Table 1, S3 Table). Of these, only 33 saliva samples provided usable results. The 33 saliva samples were from patients suffering from mild (n = 4), moderate (n = 10), or severe (n = 6) OSA and from rhonchopathy patients (n = 4) as well as from 9 healthy controls (n = 9).

For TFF2 analysis in saliva, the mean age was 50 yrs (55 yrs mild; 51.6 yrs moderate; 54.2 yrs severe; 37.8 yrs rhonchopathy; 42.8 yrs controls) and the median BMI was 28.6 kg/m^2^ (26.6 kg/m^2^ mild; 29.2 kg/m^2^ moderate; 29.4 kg/m^2^ severe; 28.4 kg/m^2^ rhonchopathy; 24.4 kg/m^2^ controls) (S4 Table). Of these, 12 were smokers and 21 were non-smokers.

### Immunohistochemical analysis

Immunohistochemistry was performed with paraffin sections (5 μm) from 15 patients (S5 Table). The slides were deparaffinized in xylene and rehydrated in a decreasing alcohol series. After placement in aqua dest., the slides were incubated in 3% H_2_0_2_ for 10 minutes, then washed three times in aqua dest. and boiled in citrate buffer (pH 6) for 10 minutes. After cooling for 1 h in citrate buffer, another washing step with aqua dest. and TBST followed. The slides were subsequently incubated in 5% normal rabbit serum for TFF3 and -2 (Dako, Glostrup, Denmark A/S) for 20 minutes and blocked with the Avidin-Biotin Blocking Kit (ThermoFisher Invitrogen, Waltham, MA, USA). After washing the slides with TBST, immunohistochemical staining was done with antibodies to mouse anti-TFF3 (1:100, Santa Cruz Biotechnology, Dallas, Texas, USA) and goat anti-TFF2 (1:100, Santa Cruz Biotechnology, Dallas, Texas, USA) at 4°C overnight. Slices were washed with TBST again, followed by incubation with secondary antibody (TFF3) rabbit anti mouse (1:200 Dako Denmark) and (for TFF2) rabbit anti goat (1:200 Dako, Denmark) for 1 hour. In between the subsequent steps slices were washed with TBST three times in each case: after the secondary antibody, incubation followed with ABC (Vector, Burlingame, USA) for 30 minutes and AEC solution (Dako, North America Inc, Carpinteria, Carlifornia, USA) for 2 minutes. After another washing step with aqua dest., Meyer’s hematoxylin was used for counterstaining. In the final step, the slides were fixed with Aquatex (Merck, Darmstadt, Germany) and examined under a 9000E Keyence microscope.

### RNA isolation and RT-PCR

For RNA isolation, 7 samples with mild (n = 2), moderate (n = 2), severe OSA (n = 1) and with rhonchopathy (n = 2) were chosen. RNA was isolated as described in [26]. Tissue was pulverized in the SpeedMill and TRIzol and chloroform were added. Then centrifugation separated the different phases. After transferring the produced supernatant to another test tube, the same amount of isopropanol was added, inducing RNA precipitation. The RNA pellet was dissolved with DEPC H_2_O. Finally, the RNA concentration was determined.

2 μg total RNA was used for complementary cDNA synthesis following the manufacturer’s protocol (RevertAid H Minus Reverse Transcriptase, Fermentas, ThermoFisher, Waltham, MA, USA). Using 5’-GTG CCA GCC AAG GAC AG-3’ (TFF3s) and 5’-CGT TAA GAC ATC AGG CTC CAG-3’ (TFF3 as) as primer, reverse transcription and amplification were performed. The PCR reaction included 42 cycles: heating to 95°C for 5 minutes, 40 cycles at 95°C for 30 seconds, 60°C for 40 seconds, 72°C for 40 seconds and, as the final elongation step, at 72°C for 5 minutes. To exclude contamination, lung was used as positive control, and ß-actin (as housekeeping gene) served as the control for the integrity of translated cDNA.

### Protein extraction and Western blot analysis

For protein isolation, 4 tissue samples were chosen, one from each OSA severity type and one from ronchopathy. A lysis tube was prepared for each severity type. A 100 mg frozen tissue sample of the respective severity type was inserted into each lysis tube. After that, 300 μl triton buffer with 15 μl protease and phosphatase inhibitor were added. This was followed by mixing, mincing and incubation on ice for 30 minutes. The samples were subsequently centrifuged for 20 minutes. The supernatant was transferred to another test tube. Finally, extinction was measured by Bradford assay using a photometer. 40 μg of sample were loaded on 15% SDS acrylamide gel and separated by electrophoresis. Proteins were transferred to the nitrocellulose membrane by electroblotting (Semidryblot, Roth, Karlsruhe, Germany). The primary antibody serum for mouse anti-TFF3 (1:50) and GAPDH (1:1000) as control was added and stored overnight at 4°C. After washing, incubation with secondary antibody serum mouse horseradish peroxidase for TFF3 (1:5000) and GAPDH (1:10000) for 1 hour followed. Photoreaction was observed after washing and adding ECL substrate (Millipore, Darmstadt, Germany). Lung tissue served as the positive control. TFF2 was not examined by Western blot.

### ELISA

All saliva samples (n = 99) were used for ELISA and processed according to the instructions of Cloud-Clone Corp. (Wuhan, China). The saliva samples and aqua dest. as control were added to the pre-coated 96-well standard strip plate (antibodies specific for TFF3 or TFF2, respectively). Measurements were performed at 450 nm and 490 nm (TFF3) as well as 405 nm and 450 nm (TFF2). Detection of both was within the expected range of 125–8.000 pg/ml.

### Statistical analysis

Data are shown as mean ± SEM. After normal distribution and homogeneity of variance had been confirmed, the statistical significance was evaluated. To determine the influence between the OSA level and TFF3 or TFF2 we performed an analysis of variance (ANOVA) and a two-sample t test with equal variances to determine whether there was a significant mean difference between the OSA levels for TFF3 or TFF2. P-values less than 0.005 were considered statistically significant. The correlation between TFF3 and -2 protein concentrations and the OSA severity index was examined first with Pearson’s correlation. Afterwards the correlation of TFF and typical predisposing factors of OSA, such as BMI, smoking, age, sex and Epworth Sleepiness Scale (ESS), were evaluated. Finally we used a logistic regression to confirm the magnitude of the effect of TFF3 and -2 on the OSA level.

STATA12 was used for the statistical analysis of all data.

## Results

### Immunohistochemistry

**Fig 1:**
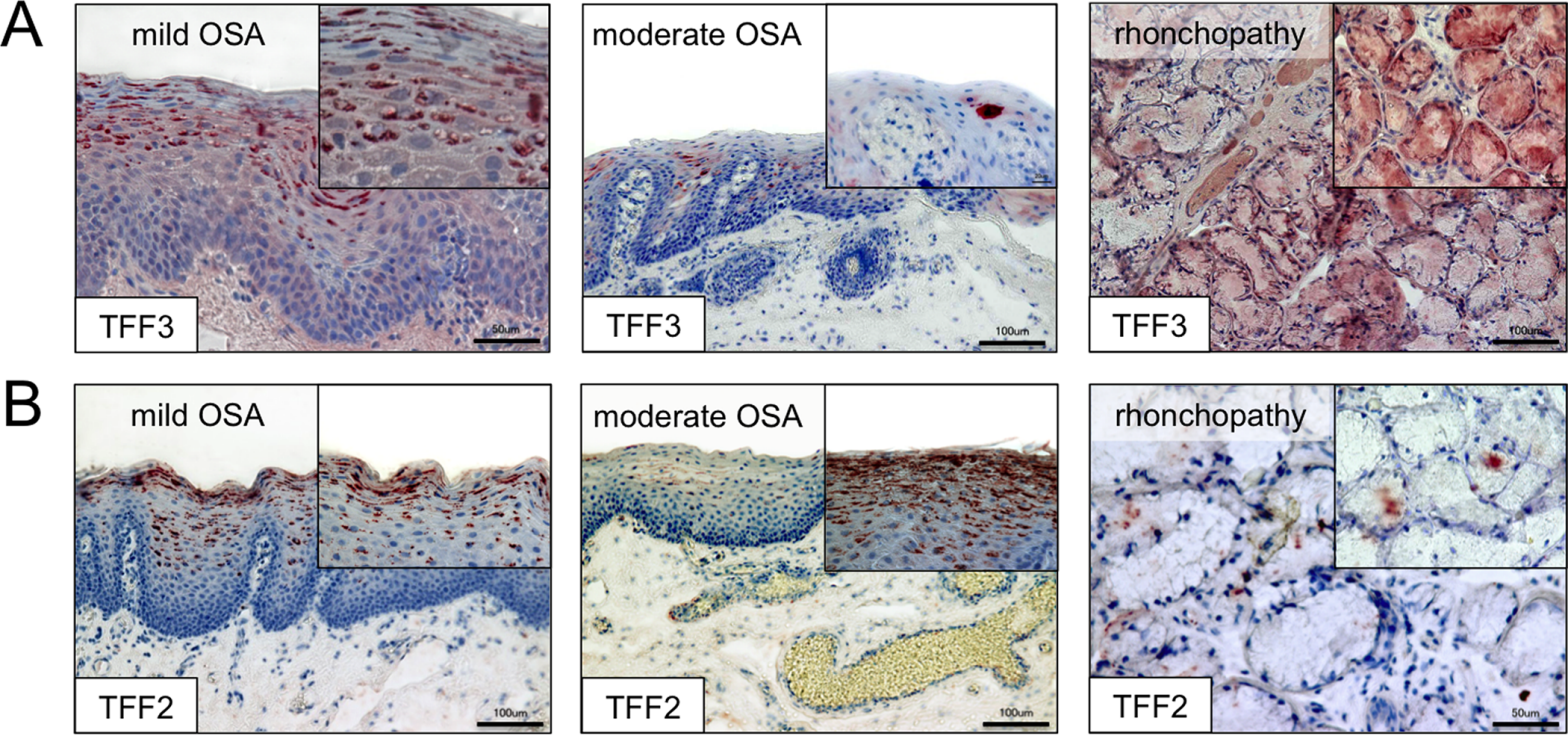
Immunohistochemical distribution of TFF3 and -2 in human uvula. (A) TFF3 immunoreactivity is visible in the epithelium of the oral side of the uvulae from patients with mild and moderate OSA as well as in the mucus glands of the uvula of patients suffering from rhonchopathy. (B) TFF2 immunoreactivity is detected in the epithelium of the oral side of uvulae from patients with mild and moderate OSA as well as in the mucus glands of the uvula of rhonchopathy patients. Red staining indicates positive immunoreactivity of the TFF antibodies. Insets in the figures show higher magnifications for the respective tissue. Scale bars: 100 μm.

15 paraffin-embedded sections (5 μm) each from mild (n = 5), moderate (n = 5) and severe OSA (n = 2) as well as from rhonchopathy (n = 3) were selected and used for immunohistochemistry (S3 Table). The presence of TFF3 and TFF2 was analyzed with specific antibodies. Red staining revealed positive reactivity of the antibodies to TFF3 and TFF2 (Fig 1A and 1B).

In mild OSA, TFF3 was found in the stratum spinosum and superficial layers of the uvula epithelium (Fig 1A). The subepithelial glands did not show reactivity to the anti TFF3 antibody. In moderate OSA, TFF3 was also visible in the upper epithelial layers, albeit subjectively with less intensity. Here, too, the subepithelial glands did not reveal any positive reactivity. No reactivity was found in severe OSA, either. In cases of rhonchopathy, TFF3 was detected in the subepithelial glands and only weak reactivity was visible in the epithelium lining of the uvula.

**Fig 2:**
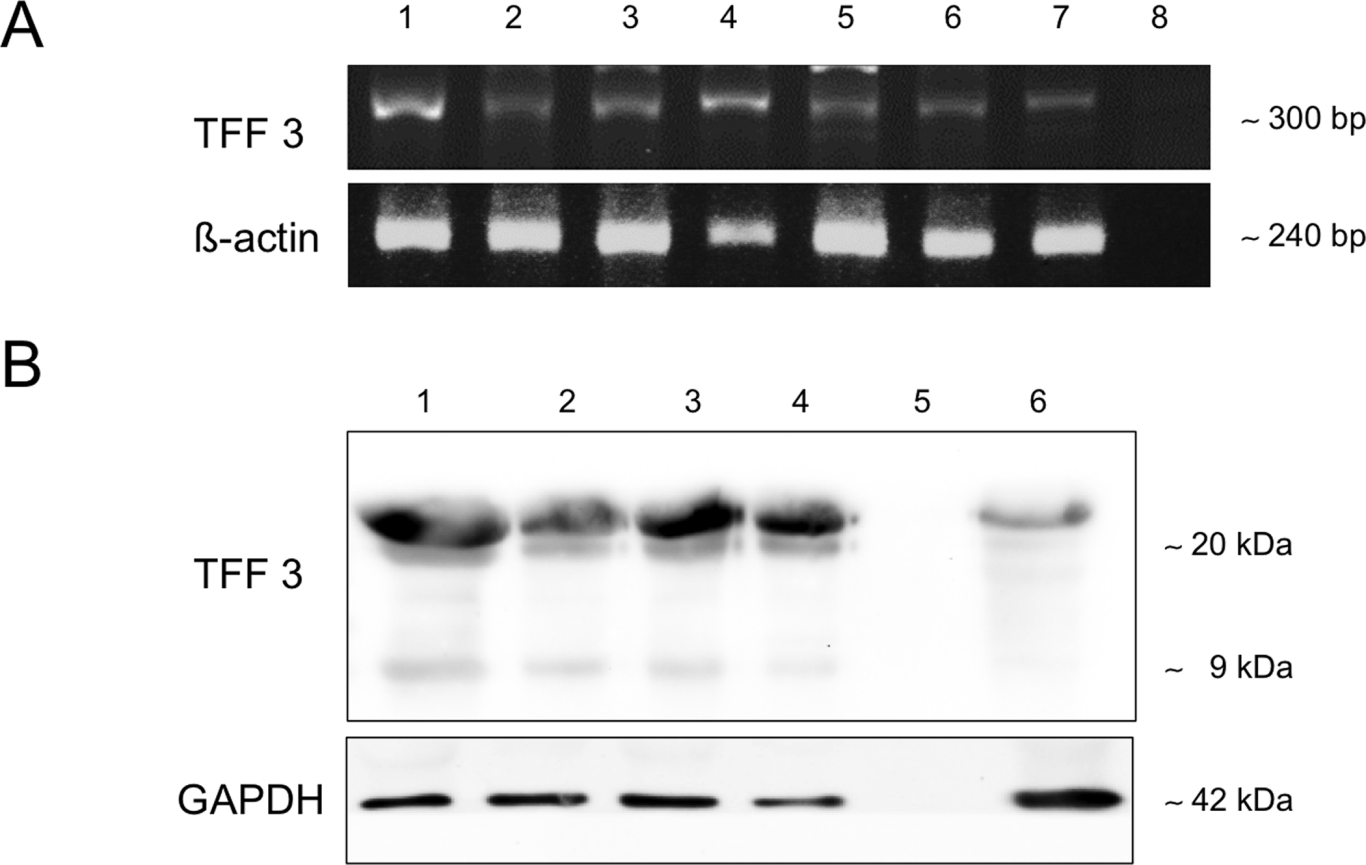
**TFF analysis by RT-PCR (A) and Western blot (B).** (A), Detection of TFF3 at ∼300 bp and ß-actin as loading control at ∼240 bp. Different samples of uvula tissue from mild OSA (lanes 1 and 2), moderate OSA (lanes 3 and 4), severe OSA (lane 5) and rhonchopathy (lane 6) are shown. DEPC-H_2_O served as negative control (lane 8), RNA-extract from lung tissue was used as a positive control (lane 7). (B) Detection of TFF3 after SDS gel electrophoresis under reducing conditions in protein isolates from the uvula tissue of patients suffering from mild OSA (lane 1), moderate OSA (lane 2), severe OSA (lane 3) and rhonchopathy (lane 4). Lung (6) served as positive control. Bands are visible at 20 kDa and 9 kDa for TFF3 and at 42 kDa for the loading control GAPDH.

Immunohistochemistry with an anti-TFF2 antibody in mild OSA revealed reactivity in the superficial epithelial cells but subjectively with less intensity when compared with TFF3 (Fig 2B). Reactivity for TFF2 was not detected in the subepithelial glands. In the epithelium of the uvula from patients with moderate OSA, anti-TFF2 antibody revealed reactivity in the superficial epithelial cells as well. The subepithelial mucous glands did not show any reactivity. In cases of severe OSA no staining was found either. However, reactivity to TFF2 was detectable in the subepithelial glands of samples from rhonchopathy cases.

### RT-PCR

RNA was isolated from 7 uvula samples after UPPP to investigate TFF3 expression. TFF3 was detected in samples of mild (n = 2), moderate (n = 2), severe OSA (n = 1) and rhonchopathy (n = 2) (Fig 2A). All samples revealed a signal at around 300 base pairs (Fig 2A). Lung tissue served as the positive control (Fig 2A). DNA bands of rhonchopathy and the different OSA severity codes were compared to ß-actin, which was positive for all samples analyzed. ß-actin, used as the loading control, confirmed homogeny of TFF3 expression in all samples (Fig 2A). Visible bands (≈ 300 bp) in RT-PCR matched with the expected sequences of human TFF3 shown in the NCBI gene bank data [27].

### Western blot analysis

After protein extraction from 4 human uvula samples with mild (n = 1), moderate (n = 1), severe OSA (n = 1) and rhonchopathy (n = 1), TFF3 was analyzed using a specific antibody to TFF3 under reducing conditions. Lung was used as the positive control (Fig 2B).

All OSA samples (mild, moderate and severe), as well as the rhonchopathy sample, showed bands at about 20 kDa and about 9 kDa (Fig 2B).

The expression pattern of TFF3 in human lung was comparable to the OSA samples and rhonchopathy sample at 20 kDa. GAPDH served as the loading control and was detected at around 42 kDa (Fig 2B).

### ELISA

Correlation between TFF3 and TFF2 protein concentrations and OSA severity (mild, moderate, severe) was investigated first. Altogether, 99 saliva samples were deconstructed in ELISA (S1 and S2 Tables). Of 99 samples, 29 had to be dropped because of measurement errors. 70 samples (n = 70) of 5 groups: mild, moderate and severe OSA, rhonchopathy and healthy controls.

The analysis of variance (ANOVA) in OSA levels showed that some variance could be explained by the influence of TFF3 and -2. This was still the case if we considered the effects of the other variables (e.g. smoking, BMI etc.).

**Fig 3:**
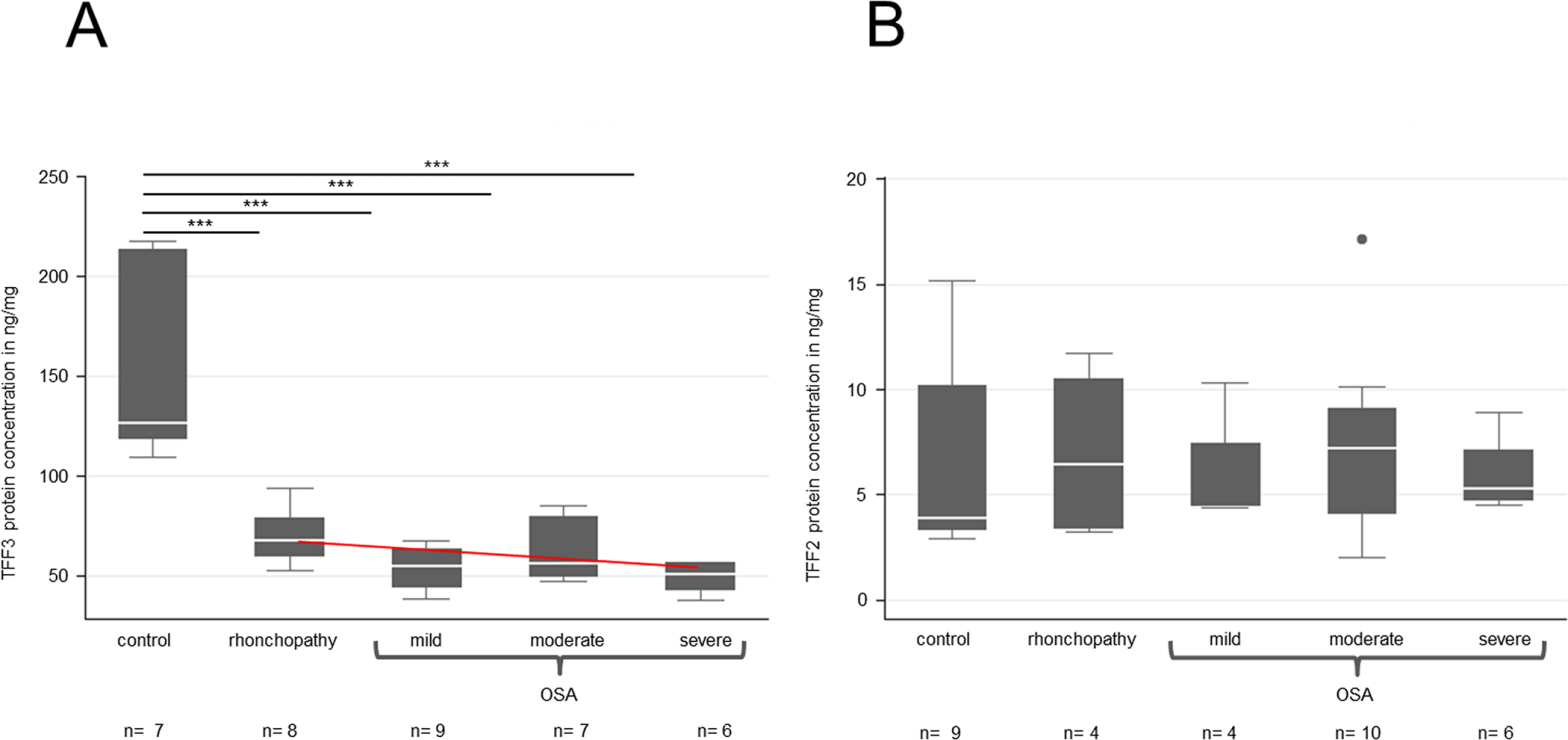
ELISA protein quantification of TFF3 and -2 in saliva samples from healthy volunteers and from patients with rhonchopathy and mild, moderate or severe OSA. (A) A significant decline in the TFF3 protein concentration is visible in all samples from patients in relation to the healthy control group without rhonchopathy. A slight but not significant fall in the protein concentrations between rhonchopathy and the different forms of OSA is visible (red line). (B) No significant differences are detectable between saliva from healthy volunteers and patients (independent of the disease or disease severity) with regard to TFF2 protein concentration.

**TFF3 protein levels** ranged from 38.1–217.8 ng/mg over all groups. For TFF3 the mean protein concentration in the healthy control group was 158 ng/mg. The mean for the rhonchopathy group was 70 ng/mg. In mild, moderate and severe OSA, the means were similar, ranging from 49.5 to 62.5 ng/mg (S4 Table). There was a significant decline in TFF3 protein concentration in relation to the control group. The decline between control and rhonchopathy was 88.0 ng/mg and between control and combined OSA groups 102.9 ng/mg (Fig 3A).

Logistic regression of the OSA levels showed a significant effect of the TFF3 concentration (p<0.05). This effect indicated a correlation between the TFF3 concentration and the OSA level although some of the control variables (sex, smoking and age) had to be dropped because they were not statistically significant (> 60%). A reason for this could be the small amount of samples investigated.

Overall, the **protein concentration of TFF2** was clearly lower than the protein concentration of TFF3 at 2–17.2 ng/mg (Fig 3B). The TFF2 protein concentration did not show any significant changes in relation to the healthy control group. The control group mean revealed a mean TFF2 protein concentration of 6.8 ng/mg. The mean of rhonchopathy was 7.0 ng/mg, and combined OSA groups showed comparable mean protein concentrations of 5.9–7.4 ng/mg (S5 Table).

The logistic regression between OSA levels and TFF2 showed no significant effects.

The correlation between protein concentrations and factors such as BMI, smoking, age, sex and ESS was then analyzed. Results from healthy controls are not shown in graphs for reasons of better clarity and comprehensibility. Because of non-response for certain variables in healthy controls in terms of TFF3, the number of observations in the tables may vary.

**Fig 4:**
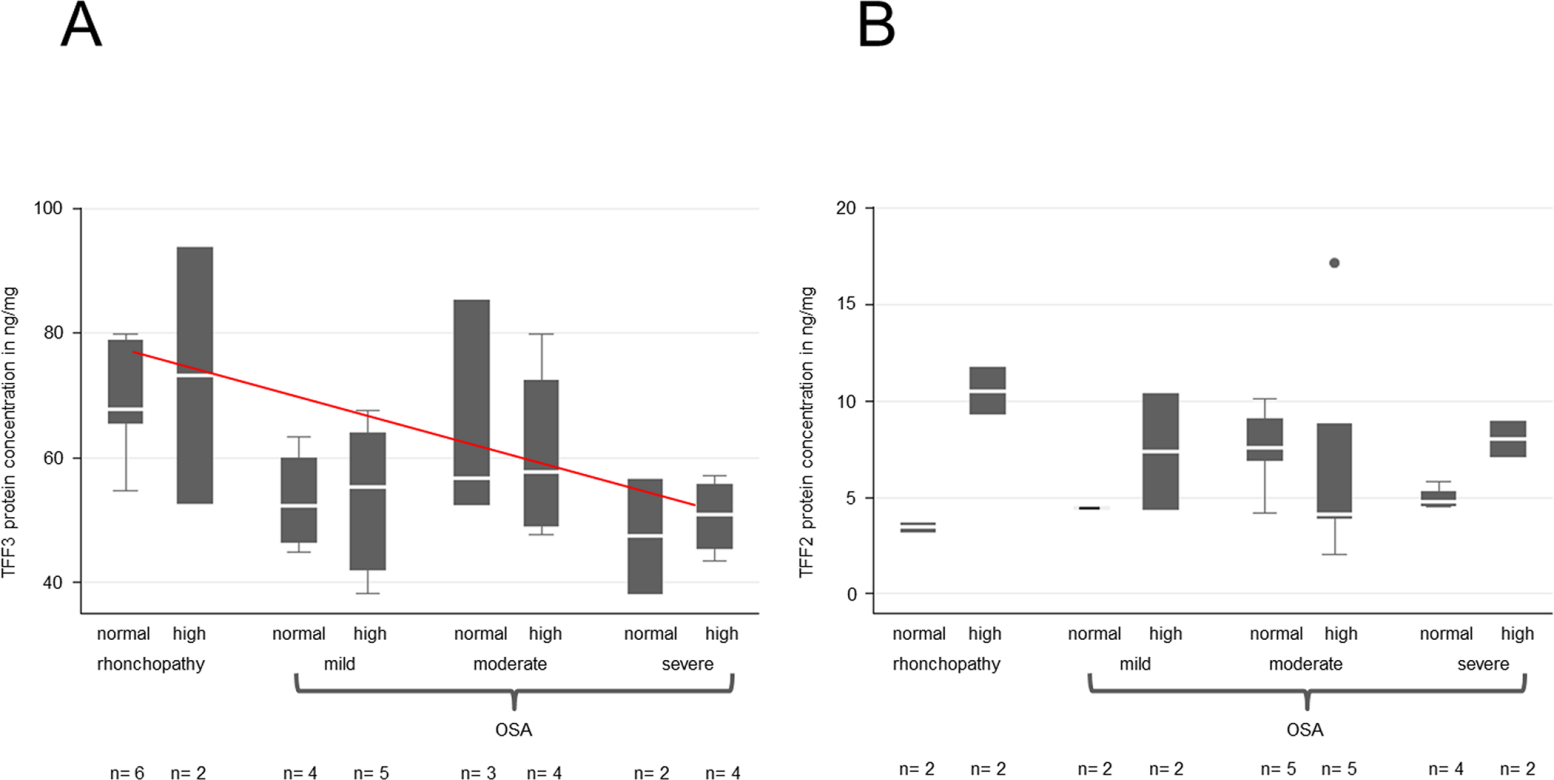
ELISA quantification of TFF3 and -2 in terms of BMI correlation in saliva samples from patients with rhonchopathy and mild, moderate or severe OSA. (A) The TFF3 protein concentration reveals a tendency (red line) to decline from rhonchopathy to severe OSA. However, this concentration decrease is not significant. (B) The TFF2 protein concentration reveals no significant changes between the different groups evaluated. In order to match patients in groups, we used the BMI classification of Diet and Health depending on age: 
Age                BMI19–24 years            19-2423–34 years            20-2535–44 years            21-2645–54 years            22-2755–64 years            23-28>65 years            24-29 [28]

**Fig 5:**
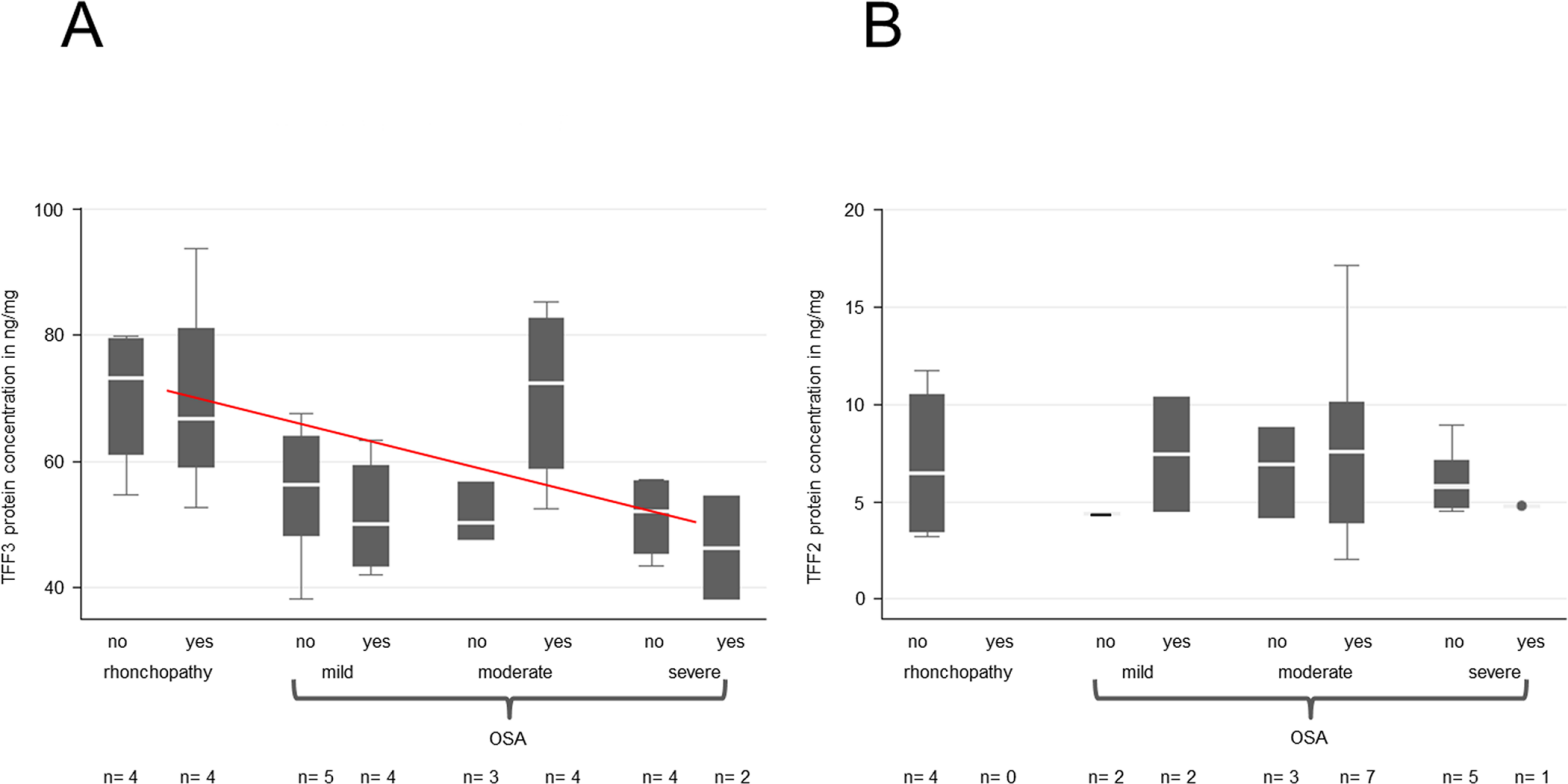
ELISA quantification of TFF3 and -2 concentrations with regard to smoking in saliva samples from patients with rhonchopathy and mild, moderate or severe OSA. (A) The TFF3 protein concentration reveals a tendency to decline between non-smokers and smokers from rhonchopathy to severe OSA (red line). However, this concentration decrease is not significant. (B) The TFF2 protein concentration does not reveal any significant changes.

**Fig 6:**
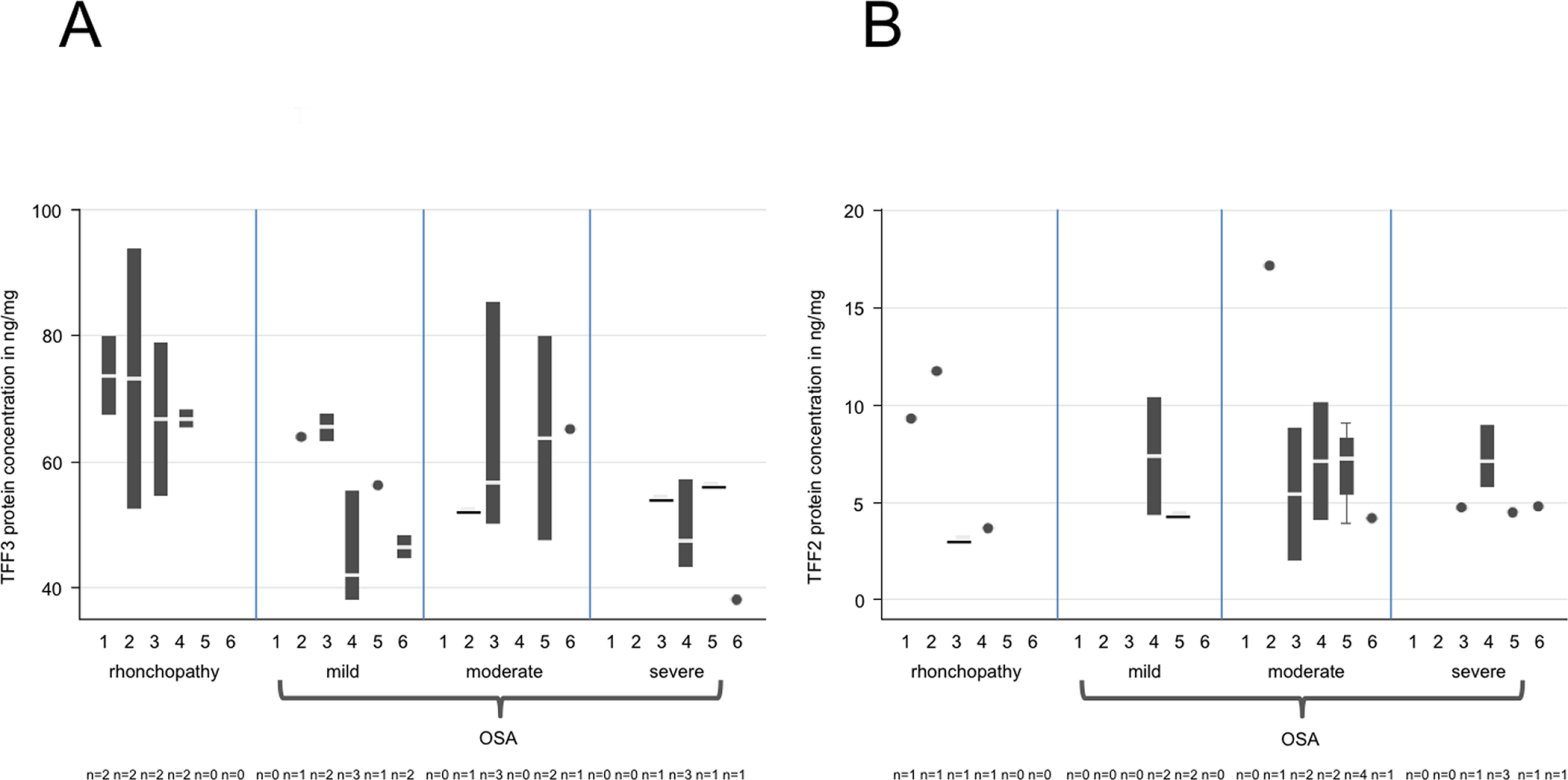
ELISA quantification of TFF3 and -2 protein concentration with regard to age in saliva samples from patients with rhonchopathy and mild, moderate or severe OSA. No significant changes are observed between the different groups evaluated for TFF3 (A) or TFF2 (B). We defined six age groups:
Group 1 = 19–24 yearsGroup 2 = 25–34 yearsGroup 3 = 35–44 yearsGroup 4 = 45–54 yearsGroup 5 = 55–64 yearsGroup 6 = >65 years

**Fig 7:**
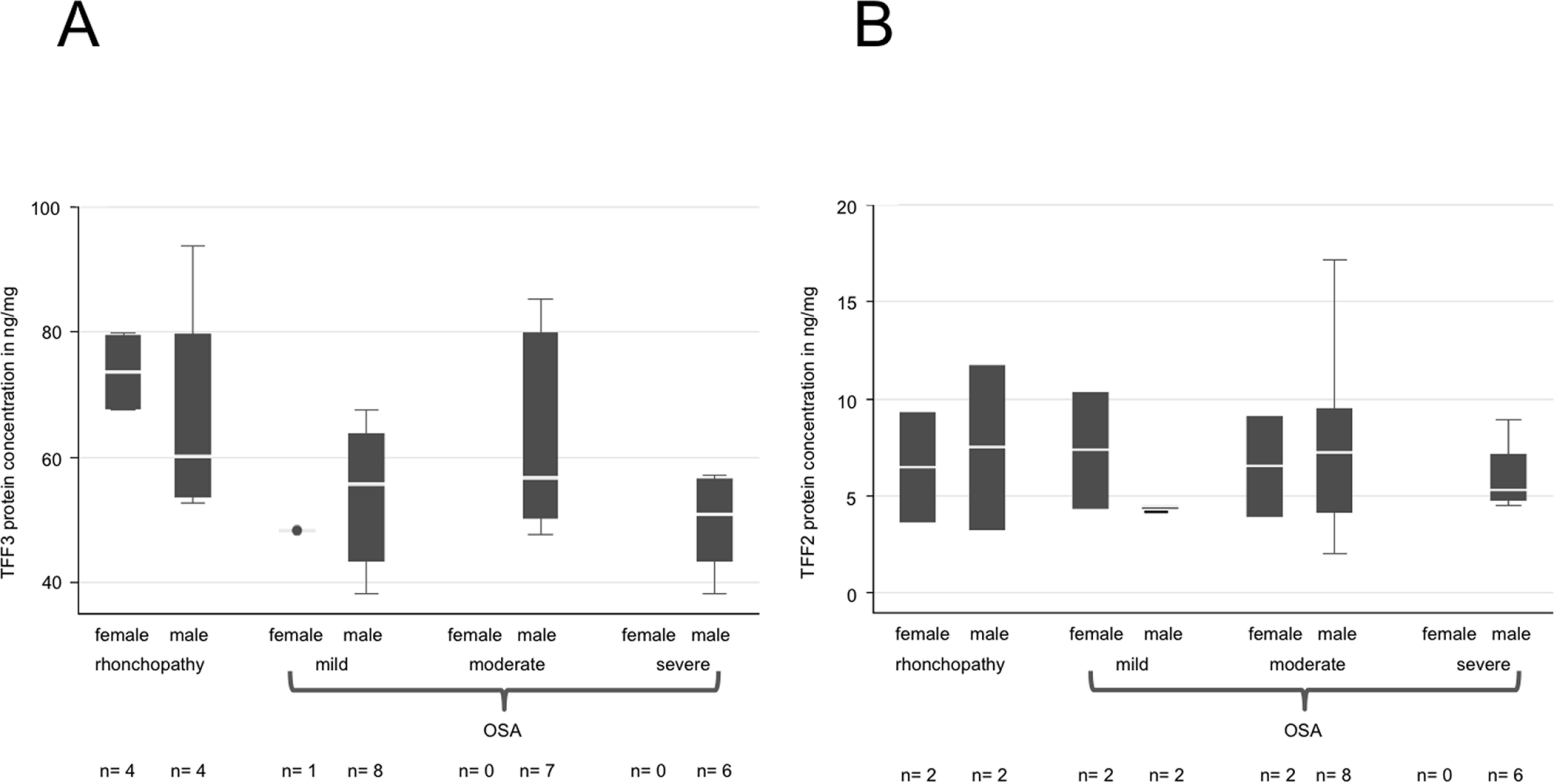
ELISA quantification of TFF3 and -2 protein concentration with regard to gender in saliva samples from patients with rhonchopathy and mild, moderate or severe OSA. No significant changes can be observed between the different groups evaluated for TFF3 (A) or TFF2 (B).

**Fig 8:**
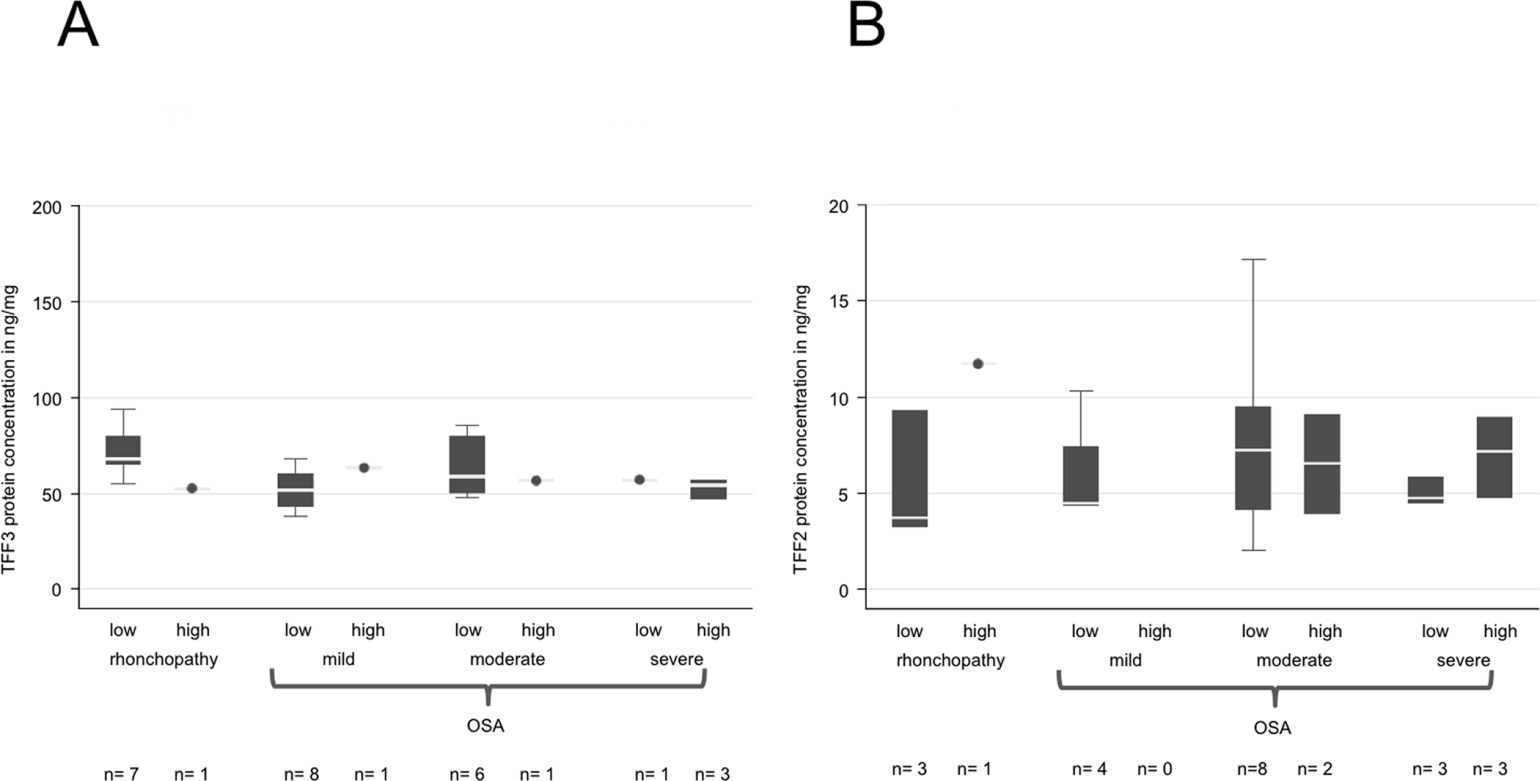
ELISA quantification of TFF3 and -2 protein concentration with regard to the Epworth Sleepiness Scale (ESS) in saliva samples from patients with rhonchopathy and mild, moderate or severe OSA. No significant changes are observed between the different groups evaluated for TFF3 (A) or TFF2 (B). The Epworth Sleepiness Scale assessment is based on a standardized questionnaire that retrospectively measures the probability of falling asleep in typical daily situations. The subjective assessment is done by patients who can choose between a scale of 4 probabilities (0 = never falling asleep to 3 = high probability of falling asleep). The sum results in a code distinguishing between low ESS <11/24 and high ESS ≥11/24.

With regard to BMI, smoking, age, gender and ESS-scores no significant changes were observed in the protein levels. (Figs 4–8)

## Discussion

OSA is a multifactorial disease in which an increase in upper airway resistance plays an important role [29, 30]. In our study, we detected and localized TFF3 as well as TFF2 in the human uvulae of patients with mild, moderate and severe OSA and of patients with rhonchopathy. Positive antibody reactivity was detected in the epithelia and to some extent in the mucous glands of the uvula. Semiquantitatively, the immunoreactivity of TFF3 and TFF2 decreased with an increasing OSA severity code. Expression of TFF3 has already been demonstrated in the large salivary glands, especially the submandibular and sublingual glands, as well as in the small salivary glands [20]. Moreover, TFF peptides are also expressed in other oral tissues such as oral epithelium and gingiva [20, 31, 32] and occur in saliva (46). Chaiyarit and co-workers examined TFF peptides in the gingival tissues of patients with chronic periodontitis and found a significantly reduced TFF3 concentration in pathologically altered tissue samples [31, 32].

Using Western blot analysis, we detected a signal for TFF3 at around 9 kDa and a further signal at around 20 kDa under reducing conditions. The monomer form usually occurs at around 7 kDa and the dimeric form at 14 kDa [33]. Albert and co-workers found non-reduced TFF3-homodimer bands at about 20 kDa [34]. However, TFF3 hetero-dimers called fusion proteins have also been described at 20 kDa [35]. Hypothetically, this could indicate in our study that TFF3 most likely aggregates with mucins and forms heterodimeric structures. As demonstrated for several other tissues and conditions, for example cornea [36] or experimentally-induced dry eye disease [26], TFF3 has positive effects on wound healing mechanisms (epithelial restitution), shows growth factor-like functions, and its expression is induced under inflammatory conditions. We here analyzed TFF3 expression and action in the course of OSA and rhonchopathy to determine whether the rapid healing capacity of TFF3 is also active in a mucosa that is permanently exposed to vibration trauma during sleep [20, 37]. To quantify and elucidate possible differences in the protein concentration in different severity codes of OSA, we performed ELISAs. TFF3 revealed higher total protein concentrations than TFF2 in healthy as well as in diseased human samples of the uvula. This is in line with ELISA measurements in human saliva from healthy individuals, where TFF3 is the predominant TFF peptide, followed by TFF1 and TFF2 [32, 38].

No statistically significant change in the TFF3 and TFF2 protein concentration was observed in correlation to factors for OSA such as BMI, smoking, age, sex or the Epworth Sleepiness Scale (ESS). In active smokers, TFF3 has been shown to be overexpressed in bronchial epithelium and in non-inflamed wild-type colon mucosa when exposed to nicotine [39, 40]. In contrast to our results, positive correlations between TFF3 and -2 expression and age have been observed in healthy children compared to children suffering from mucositis [41]. Another study described decreasing TFF3 mRNA expression in the kidneys during aging [42]. However, these data were collected in a rat model. With regard to gender, it has been described that increasing TFF3 and -2 serum levels, as well as cyclical changes in the TFF3 levels of cervical mucus [43, 44], occur in females during pregnancy.

In contrast to the published literature, where most studies that investigate TFF3 in relation to a certain disease reveal an increase in the TFF3 concentration, we found a significant reduction in the TFF3 concentration in OSA and rhonchopathy samples in relation to the healthy control group. This is consistent with studies on the oral mucosa of patients with chronic periodontitis or oral squamous cell carcinoma [31, 32]. The authors speculate that, in chronic periodontitis, bacteria may influence the TFF3 expression negatively. For TFF2 their results indicated no significant differences compared to the control group, which is also in line with our results. However, no differences in the severity of the disease (chronic periodontitis) were revealed by analysis [31].

TFFs were first investigated in cases of ulcerative conditions in the gastrointestinal tract, where they are expressed and protect the epithelial barrier from a variety of assaults [45, 46] together with mucins. Mice that lack Tff3 (Tff3 KO mice) show impaired wound healing capacity [47]. Wiede et al. discuss a structural and protective function of TFF3 for the airway mucus, whereby TFF3 probably interacts with the mucins MUC5B and MUC5AC [48]. To date it is generally accepted that the dimeric isoform of TFF3, more than its monomeric isoform, interacts directly with mucins, increasing the viscosity and elasticity of mucin-containing fluids [19]. It has been hypothesized that this may be their leading mechanism of action. The motogenic effects of TFF3 play an important role during epithelial restitution and ensure rapid sealing of the epithelial layer following injuries [36].

Our results suggest an involvement of TFF3 in the pathogenesis of OSA. Predisposing factors of OSA like BMI or age do not show a correlation with TFF3. TFF2 does not seem to correlate with OSA at all. Vibration trauma leads to structural changes in the uvula, including the epithelium [14]. These changes seem to be associated with a significant reduction in TFF3 production by the epithelium and subepithelial mucous glands, whereas no changes occur with regard to TFF2 production. We can only speculate that the continuous epithelial and subepithelial traumata that occur in cases of rhonchopathy and OSA result in a reduced production of TFF3 that is “normally”, under different circumstances (non-continuous trauma or non-chronic trauma), upregulated in mucosa and promotes epithelial restitution. Although the TFF2 concentration does not change in rhonchopathy and OSA, the reduction in TFF3 concentration could be associated with structural changes in mucus organization, leading to an increase in mucus viscosity and hence to increased breathing resistance. This vicious circle would contribute to the increased collapsibility of the upper airways in OSA patients.

Kirkness and co-workers treated OSA patients with surfactants to lower the surface tension of the fluid film lining the mucosa. They concluded that the addition of surfactants was helpful in mild intensities [4]. Comparable studies were performed by Jokic and co-workers, who were able to reduce the severity of mild and moderate OSA using this approach [49, 50]. Data from a phase II multicenter, randomized placebo-controlled trial of the prophylactic effects of recombinant human TFF3 (rhTFF) in treatment of chemotherapy-induced oral mucositis indicated that rhTFF3 in an oral spray formulation is a safe and well-tolerated drug when given concurrently with chemotherapy. Prophylactic use of rhTFF3 at either a high or a low dosage was associated with a significant reduction (≈80%) in the occurrence of mucositis in patients at high risk of progression of the lesion [51]. As rhonchopathy and OSA, like mucositis, have an inflammatory component, these results suggest the need for further elucidation of the function and effects of rhTFF3 in the oral cavity in cases of rhonchopathy and OSA within the framework of the development of novel treatment strategies.

Further investigations on the possible role of TFF1 and changes in specific mucins in cases of rhonchopathy and OSA, as well as the influence of TFF3 on mucus rheology and surface tension of normal mucus and OSA cases will be of great interest in terms of obtaining deeper insight into the pathophysiological processes that occur in OSA as well as the development of new treatment options. Assessment of the chemokine receptors CXCR4 and CXCR7 would be of interest in this context. It has been demonstrated that dimers of CXCR4 and CXCR7 are involved in TFF3-dependent activation of cell migration, but not cell proliferation [52]. These results suggest a dependence of TFF3 activity in cell migration regulated by the chemokine receptors CXCR4 and CXCR7 (at least at the ocular surface).

As TFF3 has also been successfully used in the treatment of oral mucositis in cancer patients (59, 62), an attempt to treat cases of OSA with recombinant human TFF3 would appear promising.

The present study is limited by its descriptive approach as well as the small sample size that could be used for each method applied and for the patient subgroups created. Nevertheless, the study shows that TFF3 is negatively correlated with OSA and rhonchopathy which is in contrast to most studies published so far, whereas TFF2 shows no correlation. It is of interest that risk factors of OSA or rhonchopathy do not show any correlation with TFF3 or with TFF2 protein levels.

## Supporting information

S1 Table Data of patients with mild, moderate or severe OSA, with rhonchopathy and healthy controls for TFF3. (PDF)

S2 Table Data of means and standard deviation and distribution of gender and smoking in percent for TFF3. (PDF)

S3 Table Data of patients with mild, moderate or severe OSA, with rhonchopathy and healthy controls for TFF2. (PDF)

S4 Table Data of means and standard deviation and distribution of gender and smoking in percent for TFF2. (PDF)

S5 Table Data of means and standard deviation and distribution of gender and smoking in percent for TFF2. (PDF)

## Abbreviations

(RT)-PCR: (Reverse Transcriptase) Polymerase Chain Reaction
°C: Grad Celsius
AASM: American Academy of Sleep Medicine
ABC: Avidin Biotin Complex
AEC: Aminoethyl Carbazole
AHI: Apnea Hypopnea Inde
aqua dest.: Aqua destillated
ASA: American Society of Anesthesiologists
BIS: Bispectral Analysis
BMI: Body Mass Index
bp: Base Pair
cDNA: Complementary Deoxyribonucleic Acid
(non) CPAP: (Non-) Continuous positive airway pressure
CXCR (4, 7): C-X-C chemokine receptor type (4,7)
DEPC- H_2_O: Diethylpyrocarbonate- water
DGSM: Deutsche Gesellschaft für Schlafforschung und Schlafmedizin
DIESE: Drug-induced Sleep Endoscopy
ECG: Electrocardiogram
ECL: Enhanced Chemiluminescence
EEG: Electrooculogram
ELISA: Enzyme Linked Immunosorbent Assay
ESS: Eppworth Sleepiness Scale
GAPDH: Glycerinaldehyde 3-phosphate dehydrogenase
h: Hour
IHC: Immunohistochemistry
kDa: Kilo Dalton
KO mice: Knockout mice
mg: Milligram
ml: Milliliter
MUC (2, 5AC, 5B, 7): Mucin (2, 5AC, 5B, 7)
n: Amount
NCBI: National Center for Biotechnology Information
ng: Nanogram
nm: Nanometer
ORL: Otorhinolaryngology
OSA: Obstructive sleep apnea syndrome
pg: Picogram
PSG: Polysomnography
rhTFF: Recombinant human Trefoil Factor Family
RNA: Ribonucleic acid
SDS: Sodium Dodecyl Sulfate
SEM: Standard Error of Mean
std.dev.: Standard deviation
TBST: Tris-buffered saline with Tween
TCI: Target Controlled Infusion
TFF (1,2,3): Trefoil factor family peptides (1,2,3)
μg: Microgram
μl: Microliter
UPPP: Uvulopalatopharyngoplasty
y: year
