# Supplementary material for: Obstructive sleep apnea and rhonchopathy are associated with downregulation of trefoil factor family peptide 3 (TFF3)—Implications of changes in oral mucus composition
Source: PLoS One. 2017 Oct 13;12(10):e0185200. doi: 10.1371/journal.pone.0185200 (PMC5640215; doi:10.1371/journal.pone.0185200)
Supplement: S1 Table — (PDF) [file pone.0185200.s001.pdf]

Supplement table 1– Data of patients with mild, moderate or severe OSA, with rhonchopathy and healthy controls for TFF3.

Group A (TFF3 saliva, mild OSA)

Patient*	Age (*yr*)	BMI (*kg/m²*)	sex	AHI	ESS	smoking
1	70	32.2	m	13.2	12	yes
2	63	27.1	f	12.6	2	yes
3	49	29.7	m	8.4	8	yes
4	38	25.2	m	13.6	12	yes
5	52	27.4	m	7.0	11	no
6	75	25.6	f	10.4	2	no
7	30	28.3	m	6.2	8	no
8	63	25.0	m	5.9	10	no
9	53	30.6	m	11.3	3	yes
10	43	27.1	m	14.0	11	no
11	55	25.2	f	10.0	4	no
12	72	21.5	m	10.1	5	yes

Group B (TFF3 saliva, moderate OSA)

Patient*	Age (*yr*)	BMI (*kg/m²*)	sex	AHI	ESS	smoking
1	35	25.8	m	22.3	6	no
2	72	35.0	m	26.3	0	yes
3	50	40.4	f	24.4	14	yes
4	57	28.8	m	27.1	7	no
5	57	32.3	m	17.9	8	yes
6	51	36.8	m	23.3	4	no
7	43	24.3	m	18.2	14	no
8	34	24.7	m	26.2	10	yes
9	36	21.0	m	15.6	10	yes
10	49	38.0	f	23.5	18	yes
11	66	27.2	m	21.0	14	yes

Group C (TFF3 saliva, severe OSA)

Patient*	Age (*yr*)	BMI (*kg/m²*)	sex	AHI	ESS	smoking
1	51	36.4	m	66.3	11	yes
2	52	35.4	m	57.7	5	yes
3	50	31.9	m	38.0	9	no
4	60	26.7	m	47.2	16	no
5	83	24.1	m	51.3	1	yes
6	52	30.0	f	53.0	7	yes
7	66	29.1	m	39.4	4	yes
8	53	48.6	m	62.0	13	no
9	43	37.5	m	108.1	13	yes
10	46	27.5	m	75.3	8	no

Group G (TFF3 saliva, rhonchopathy)

Patient*	Age (*yr*)	BMI (*kg/m*^{2})	sex	AHI	ESS	smoking
1	49	22.9	m	< 5	5	yes
2	54	25.5	m	< 5	7	yes
3	53	23.3	f	< 5	7	yes
4	22	19.6	f	< 5	6	no
5	23	23.6	m	< 5	7	no
6	37	23.5	f	< 5	11	no
7	31	26.6	m	< 5	12	yes
8	20	22.1	f	< 5	4	no
9	40	19.7	m	< 5	7	no
10	30	25.8	m	< 5	9	yes
11	54	25.0	m	< 5	9	no

Group H (TFF, saliva, controls/healthy)

Patient*	Age (yr)	BMI (kg/m2)	sex	AHI	ESS	smoking
1	23	18.1	f	< 5	3	no
2	n/a	n/a	f	< 5	n/a	n/a
3	29	19.3	f	< 5	n/a	no
4	25	24.8	m	< 5	0	no
5	26	25.2	m	< 5	4	no
6	43	20.8	f	< 5	11	no
7	34	23.5	f	< 5	3	no
8	28	20.5	f	< 5	10	yes
9	54	20.1	f	< 5	9	no
10	44	27.4	m	< 5	4	no
11	26	23.6	m	< 5	2	no
12	24	18.7	f	< 5	5	no
13	25	22.2	m	< 5	6	no
