# Supplementary material for: Obstructive sleep apnea and rhonchopathy are associated with downregulation of trefoil factor family peptide 3 (TFF3)—Implications of changes in oral mucus composition
Source: PLoS One. 2017 Oct 13;12(10):e0185200. doi: 10.1371/journal.pone.0185200 (PMC5640215; doi:10.1371/journal.pone.0185200)
Supplement: S2 Table — (PDF) [file pone.0185200.s002.pdf]

Supplement table 2– Data of patients with mild, moderate or severe OSA, with rhonchopathy and healthy controls for TFF2

Group A (TFF2, saliva, mild OSA)

Patient*	Age (*yr*)	BMI (*kg/m²*)	sex	AHI	ESS	smoking
1	48	29.0	m	11.7	4	no
2	54	27.1	f	13.0	5	yes
3	55	26.6	m	13.0	0	yes
4	63	23.5	f	6.6	11	no

Group B (TFF2, saliva, moderate OSA)

Patient*	Age (*yr*)	BMI (*kg/m²*)	sex	AHI	ESS	smoking
1	57	33.9	m	20.2	9	no
2	28	32.3	m	17.3	4	yes
3	60	25.8	f	19.2	14	yes
4	55	27.4	m	21.3	6	yes
5	37	26.5	m	25.0	10	yes
6	55	30.7	m	24.0	18	yes
7	67	28.7	m	20.8	6	no
8	64	24.7	m	28.2	6	no
9	61	35.2	f	23.9	20	yes
10	48	27.1	f	19.5	8	no
11	74	23.9	m	26.2	3	no
12	46	38.3	m	18.4	3	yes
13	46	29.6	m	15.4	0	no
14	44	27.4	m	15.7	8	no
15	54	25.4	m	18.4	7	yes

Group C (TFF2, saliva, severe OSA)

Patient*	Age (*yr*)	BMI (*kg/m²*)	sex	AHI	ESS	smoking
1	51	25.2	m	34.9	1	no
2	41	25.8	m	47.7	0	no
3	52	43.9	m	77.0	4	no
4	49	33.4	m	54.2	13	no
5	52	35.5	m	118.0	20	no
6	59	27.7	m	33.0	6	no
7	56	29.2	f	31.4	6	no
8	64	44.8	m	73.9	22	no
9	73	28.8	m	32.4	14	yes

Group G (TFF2, saliva, rhonchopathy)

Patient*	Age (*yr*)	BMI (*kg/m²*)	sex	AHI	ESS	smoking
1	60	26.8	f	4.7	9	no
2	50	31.5	f	4.1	19	no
3	33	30.7	f	3.1	11	no
4	24	23.5	m	1.3	11	no

Group H (TFF2, saliva, healthy controls)

Patient*	Age (yr)	BMI (kg/m2)	sex	AHI	ESS	smoking
1	44	21.5	f	< 5	11	no
2	34	24.98	f	< 5	13	no
3	49	28.4	m	< 5	11	no
4	31	20.55	f	< 5	13	yes
5	24	18.44	f	< 5	3	no
6	28	24.38	m	< 5	2	no
7	50	30	f	< 5	2	no
8	78	29.14	f	< 5	4	no
9	53	24.38	m	< 5	3	no
10	45	26.6	m	< 5	7	yes
