# Supplementary material for: Obstructive sleep apnea and rhonchopathy are associated with downregulation of trefoil factor family peptide 3 (TFF3)—Implications of changes in oral mucus composition
Source: PLoS One. 2017 Oct 13;12(10):e0185200. doi: 10.1371/journal.pone.0185200 (PMC5640215; doi:10.1371/journal.pone.0185200)
Supplement: S3 Table — (PDF) [file pone.0185200.s003.pdf]

Supplement table 3– Data of biopsy patients with mild, moderate or severe OSA, with rhonchopathy and healthy controls.

Group A (biopsy, mild OSA)

Patient*	Age (*yr*)	BMI (kg/m^2)	sex	AHI	ESS	smoking
1	46	28.5	m	7.9	19	yes
2	41	27.2	m	11.0	17	no
3	43	28.6	m	14.0	12	no
4	55	24.0	m	12.5	7	no
5	36	33.9	f	12.0	9	no

Group B (biopsy, moderate OSA)

Patient*	Age (*yr*)	BMI (kg/m^2)	sex	AHI	ESS	smoking
1	34	24.1	m	20.1	10	yes
2	41	33.8	m	24.6	14	no
3	50	25.7	m	16.2	6	yes
4	51	36.8	m	23.3	4	no
5	27	34.7	m	28.9	9	no

Group C (biopsy, severe OSA)

Patient*	Age (*yr*)	BMI (kg/m^2)	sex	AHI	ESS	smoking
1	46	21.0	m	34.7	9	yes
2	49	30.6	m	31.6	7	yes

Group G (biopsy, rhonchopathy)

Patient*	Age (*yr*)	BMI (kg/m^2)	sex	AHI	ESS	smoking
1	53	25.4	m	< 5	0	yes
2	34	29.7	m	< 5	9	yes
3	29	33.8	m	< 5	3	yes
