# Supplementary material for: Obstructive sleep apnea and rhonchopathy are associated with downregulation of trefoil factor family peptide 3 (TFF3)—Implications of changes in oral mucus composition
Source: PLoS One. 2017 Oct 13;12(10):e0185200. doi: 10.1371/journal.pone.0185200 (PMC5640215; doi:10.1371/journal.pone.0185200)
Supplement: S4 Table — (PDF) [file pone.0185200.s004.pdf]

Supplement table 4– Data of means and standard deviation and distribution of gender and smoking in percent for TFF3

TFF3 protein concentration/ total protein

	mean	std.dev	p
control	158.1	48.8	-
rhonchopathy	70.1	13.7	0.999
mild OSA	53.3	10.5	0.999
moderate OSA	62.5	14.9	0.999
severe OSA	49.5	7.8	0.999

TFF3 by age

	mean	std.dev.
control	37.5	15.9
rhonchopathy	35.6	12.7
mild OSA	52.8	15.1
moderate OSA	47.4	14.8
severe OSA	55.7	14.2

TFF3 by ESS

	mean	std.dev.
control	4.4	3.4
rhonchopathy	7.9	2.6
mild OSA	7.8	3.7
moderate OSA	7.9	4.3
severe OSA	12.8	2.9

TFF3 by BMI

	mean	std.dev.
control	22.9	3.0
rhonchopathy	23.3	2.6
mild OSA	26.7	2.8
moderate OSA	27.5	4.9
severe OSA	32.8	9.2

TFF3 by gender

	male (%)	female (%)
control	42.9	57.1
rhonchopathy	50	50
mild	11.1	88.9
moderate	100	0
severe	100	0

TFF3 by smoking

	smoker (%)	non-smoker (%)
control	0	100
rhonchopathy	50	50
mild	44.4	55.6
moderate	57.1	42,.9
severe	33.3	66.7
