# Supplementary material for: Obstructive sleep apnea and rhonchopathy are associated with downregulation of trefoil factor family peptide 3 (TFF3)—Implications of changes in oral mucus composition
Source: PLoS One. 2017 Oct 13;12(10):e0185200. doi: 10.1371/journal.pone.0185200 (PMC5640215; doi:10.1371/journal.pone.0185200)
Supplement: S5 Table — (PDF) [file pone.0185200.s005.pdf]

Supplement table 5– Data of means and standard deviation and distribution of gender and smoking in percent for TFF2

TFF2 proteinconcentration/ total protein

	mean	std.dev	p
control	6.8	4.4	-
rhonchopathy	7.0	4.2	0.4723
mild OSA	5.9	3.0	0.6395
moderate OSA	7.4	4.3	0.3858
severe OSA	6.0	1.8	0.6619

TFF2 by age

	mean	std.dev.
control	43.8	15.7
rhonchopathy	37.8	11.6
mild OSA	55.0	6.2
moderate OSA	53.1	11.9
severe OSA	55.7	8.9

TFF2 by ESS

	mean	std.dev.
control	7.2	4.8
rhonchopathy	12.5	4.4
mild OSA	5.0	4.5
moderate OSA	8.1	5.5
severe OSA	9.0	5.5

TFF2 by BMI

	mean	std.dev.
control	24.8	3.8
rhonchopathy	28.4	4.0
mild OSA	26.6	2.3
moderate OSA	29.2	4.3
severe OSA	32.4	7.0

TFF2 by gender

	male (%)	female (%)
control	40	60
rhonchopathy	50	50
mild	50	50
moderate	80	20
severe	90	10

TFF2 by smoking

	smoker (%)	non-smoker (%)
control	80	20
rhonchopathy	100	0
mild	50	50
moderate	46.7	53.3
severe	90	10
